# Predictive Performance of the HEART Score for Acute Coronary Syndrome with Significant Coronary Stenosis in Patients with Non–ST-Elevation Acute Chest Pain

**DOI:** 10.3390/jcdd13070335

**Published:** 2026-07-16

**Authors:** André Martins, Mónica Amado, Adriana Vazão, Joana Pereira, Luís Santos, Margarida Cabral, Célia Domingues, David Durão, João Morais

**Affiliations:** 1 Unidade Local de Saúde da Região de Leiria, E.P.E., 2410-197 Leiria, Portugal; monica.amado@ulsrl.min-saude.pt (M.A.); adriana.vazao@ulsrl.min-saude.pt (A.V.); joana.r.pereira@ulsrl.min-saude.pt (J.P.); luis.santos@ulsrl.min-saude.pt (L.S.); ana.s.cabral@ulsrl.min-saude.pt (M.C.); celia.m.domingues@ulsrl.min-saude.pt (C.D.); david.durao@ulsrl.min-saude.pt (D.D.); 2 ciTechCare—Center for Innovative Care and Health Technology, Polytechnic Institute of Leiria, 2411-901 Leiria, Portugal

**Keywords:** chest pain, acute coronary syndrome, coronary artery stenosis, HEART score

## Abstract

**Background:** Chest pain is a leading cause of emergency department (ED) visits, requiring rapid identification of acute coronary syndrome (ACS). The HEART score is widely used for risk stratification, although its ability to identify ACS associated with significant coronary artery stenosis (SCS) remains incompletely studied. This study evaluates its diagnostic performance in patients with acute chest pain. **Methods:** A retrospective analysis included patients presenting to the ED with non–ST-elevation chest pain triaged as very urgent by the Manchester Triage System. Patients with ACS underwent invasive coronary angiography. Based on the final diagnosis and angiographic findings, patients were classified into two groups: (1) ACS with SCS and (2) non-ACS or ACS without SCS. The HEART score was retrospectively calculated. Diagnostic performance for ACS with SCS was assessed using sensitivity, specificity, and receiver operating characteristic curve analysis. **Results:** Of 480 patients, 34 had ACS with SCS. The HEART score demonstrated high discriminative performance (AUC = 0.956; 95% CI 0.929–0.983), with sensitivity 88.2% and specificity 87.9% at a cut-off of ≥6. **Conclusions:** The HEART score showed strong diagnostic performance for ACS with SCS in ED patients with non–ST-elevation chest pain and may support early identification of patients requiring invasive coronary evaluation.

## 1. Introduction

Chest pain is among the most frequently reported symptoms in the emergency department (ED), accounting for approximately 10% of all patient visits [1,2]. Epidemiological data indicate that ED presentations for this complaint are increasing more rapidly than overall attendances and population growth [3]. Recognizing acute coronary syndrome (ACS) within this broad spectrum of patients remains a key diagnostic challenge, particularly in the absence of typical symptoms or diagnostic electrocardiographic changes [4]. Notably, among those admitted with chest pain, only about 25% are ultimately diagnosed with ACS [5]. Conventional methods, including clinical examination, electrocardiogram (ECG), and cardiac biomarkers, are often insufficient to reliably differentiate patients with ACS from those with non-ischemic chest pain. Consequently, a range of clinical risk scores has been developed to support the stratification of individuals presenting with chest pain [6].

Developed in 2008, the HEART score was designed as a clinical tool to stratify the risk in patients with chest pain in the ED. The score comprises five elements—history, ECG, age, risk factors, and troponin (HEART)—whose integration yields a total score closely associated with the likelihood of major adverse cardiac events (MACEs). Table 1 presents the individual components along with their corresponding point allocations [7,8]. In a multicenter validation study of the HEART score [8], MACEs occurred in 0.99% of patients classified as low risk (score 0–3), 11.6% of those at intermediate risk (score 4–6), and 65.2% of patients categorized as high risk (score 7–10).

More recent studies have explored recalibration of the HEART score to contemporary high-sensitivity cardiac troponin (hs-cTn) assays, replacing conventional troponin thresholds with hs-cTn-based cut-offs. In these adaptations, troponin scoring is commonly aligned with analytical limits of detection and the 99th percentile upper reference limit, allowing a more refined stratification of myocardial injury. Such modified approaches have demonstrated improved rule-out performance and safety for early discharge strategies compared with the original conventional troponin-based HEART score, supporting the integration of hs-cTn into contemporary applications of the score in patients presenting with acute chest pain [10].

The HEART score has been extensively validated as a reliable tool for predicting short-term MACE, providing high sensitivity and negative predictive value in identifying low-risk individuals in routine emergency care [7,8,11]. Moreover, recent investigations have extended its application, examining its association with coronary anatomy and its ability to predict ACS in patients presenting with chest pain to the ED. Several studies have shown that higher HEART scores correlate with increased complexity of coronary artery disease (CAD) [12,13,14], whereas other evidence demonstrates that the score can effectively discriminate ACS within unselected ED chest pain populations [15].

Beyond general ACS prediction, the HEART score has also been evaluated for its association with significant coronary artery stenosis (SCS). Arslan et al. reported that higher HEART scores were associated with SCS on coronary computed tomography angiography in patients with suspected ACS [16], and Han et al. confirmed similar associations with obstructive CAD on invasive angiography in patients presenting with chest pain to the ED [17]. Collectively, these findings suggest that the HEART score may provide incremental value in predicting ACS specifically related to angiographically SCS. However, current evidence remains limited, highlighting the need for further investigation to robustly establish its predictive performance in this context. This is particularly relevant given the clinical importance of identifying patients with ACS and underlying anatomically significant coronary disease, which may inform early invasive evaluation and management strategies in patients with acute chest pain.

The present study was designed to evaluate the predictive value of the HEART score for ACS with SCS in a cohort of patients presenting to the ED with non–ST-elevation acute chest pain. In addition, post-discharge outcomes at 6 and 12 months were assessed as exploratory endpoints.

## 2. Materials and Methods

### 2.1. Study Design and Population

This was a retrospective, single-center study conducted at a hospital in Portugal with 24 h cardiology and interventional cardiology facilities. A consecutive series of patients aged 18 years or older who presented to the ED with chest pain between January and May 2022 was screened for eligibility. Patients triaged as very urgent according to the Manchester Triage System were considered for inclusion. Patients with chest pain and ST-segment elevation, as well as patients with chest pain of traumatic origin, were excluded. The study protocol was approved by the institutional ethics committee, and the requirement for informed consent was waived due to the retrospective nature of the study.

### 2.2. HEART Score Assessment

For each patient, the HEART score was retrospectively calculated based on clinical data obtained during the initial ED assessment, including clinical history, physical examination, ECG, and the first available high-sensitivity cardiac troponin measurement, with results available within 60 min. Troponin was incorporated into the HEART score using a high-sensitivity adapted approach, in line with previously described modifications of the HEART score. Specifically, hs-cTnI values were categorized as follows: 0 points for values below the limit of detection, 1 point for values between the limit of detection and the 99th percentile upper reference limit, and 2 points for values above the 99th percentile. Patients were stratified into low, intermediate, and high-risk categories according to the established scoring system.

### 2.3. Clinical and Angiographic Classification

Patients were classified for diagnostic purposes into the ACS group if they presented with unstable angina or non-ST-segment elevation myocardial infarction (NSTEMI), while all others were categorized as non-ACS. Evaluation of coronary anatomy was performed using invasive coronary angiography when clinically indicated. SCS was defined as a luminal narrowing of 70% or more in at least one major epicardial coronary artery. Based on these criteria, patients were divided into two groups: Group 1 included ACS patients with SCS, and Group 2 comprised ACS patients without SCS as well as all non-ACS patients.

### 2.4. Clinical Outcomes

The primary clinical outcome was the occurrence of ACS with SCS. This outcome was employed to evaluate the predictive performance of the HEART score. Post-discharge outcomes at 6 and 12 months were also collected as an exploratory secondary endpoint.

### 2.5. Data Collection

Baseline demographic and clinical characteristics, including cardiovascular risk factors and comorbidities, were retrospectively extracted from ED electronic medical records. All patients underwent ECG recording and high-sensitivity cardiac troponin I (hs-cTnI) measurement at presentation, and these data were retrospectively reviewed by two independent investigators. Coronary anatomy data from invasive cardiac catheterization were also collected whenever the procedure was performed.

### 2.6. Statistical Analysis

For descriptive analysis, categorical variables were expressed as frequencies and percentages and compared between groups using the chi-square test or Fisher’s exact test, as appropriate. Continuous variables were tested for normality using the Kolmogorov–Smirnov test and expressed as mean ± standard deviation or median with interquartile range, according to distribution. Between-group comparisons were conducted using the independent samples *t*-test for normally distributed variables or the Mann–Whitney U test for non-normally distributed variables.

The HEART score was analyzed both as a continuous and categorical variable. The continuous form was used for receiver operating characteristic (ROC) curve analysis to assess its discriminative performance for predicting ACS with SCS in the overall study population and, in a predefined sensitivity analysis, for predicting SCS among patients with confirmed ACS. Optimal cut-off values were determined using Youden’s index. The area under the curve (AUC) with 95% confidence intervals (CIs) was calculated, and sensitivity and specificity at the optimal cut-off were reported with their corresponding 95% confidence intervals (Wilson method). The categorical form was used for descriptive and comparative analyses.

All statistical analyses were performed using IBM SPSS Statistics, version 29.0 (IBM Corp., Armonk, NY, USA). A two-sided *p*-value < 0.05 was considered statistically significant.

## 3. Results

**Figure 1:**
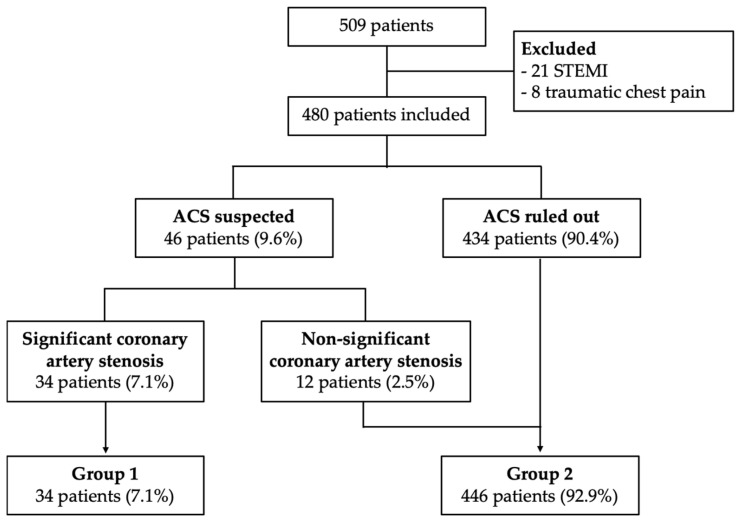
Flowchart of the study design (ACS—acute coronary syndrome, STEMI—ST-segment elevation myocardial infarction).

In 2022, 1662 patients presenting with CP were admitted to the ED and classified as very urgent according to the Manchester Triage System, corresponding to an average of five patients per day. For the present study, the first 509 consecutive patients admitted between January and May 2022 were considered. Twenty-nine patients were excluded from the analysis: 21 with ST-segment elevation myocardial infarction (STEMI) and 8 with traumatic chest pain. A total of 480 patients met the inclusion criteria and were included in the analysis. Within this cohort, ACS was suspected in 46 patients (9.6%), while 434 patients (90.4%) were classified as non-ACS. Among patients with suspected ACS, 34 (7.1%) presented with SCS on invasive coronary angiography and were assigned to Group 1. The remaining 446 patients (92.9%), including 12 ACS patients without SCS and all non-ACS patients, were allocated to Group 2 (Figure 1).

### 3.1. Patient Baseline Characteristics

Baseline patient characteristics are summarized in Table 2. The overall median age was 59 years (IQR 27), and 241 patients (50%) were male. Compared with Group 2, Group 1 patients were older (67 vs. 58 years, *p* = 0.004) and exhibited a higher prevalence of type 2 diabetes mellitus (32% vs. 17%, *p* = 0.020), dyslipidemia (74% vs. 40%, *p* < 0.001), hypertension (71% vs. 47%, *p* = 0.009), prior myocardial infarction (50% vs. 9%, *p* < 0.001), heart failure history (27% vs. 14%, *p* = 0.037) and pulmonary disease (27% vs. 8%, *p* = 0.002).

Several differences in admission characteristics were observed between groups (Table 3). Patients in Group 1 more frequently reported precordial pain (68% vs. 49%, *p* = 0.039) and pain radiating to the arm or shoulder (74% vs. 40%, *p* < 0.001), whereas pleuritic chest pain (0% vs. 11.9%, *p* = 0.023) and palpation-induced chest pain (0% vs. 13.9%, *p* = 0.014) were observed exclusively in Group 2. No significant differences were observed in the time from symptom onset to ED presentation (median 3.0 h [IQR 6.6] vs. 4.0 h [IQR 10.0], *p* = 0.369). Regarding accompanying symptoms, diaphoresis was markedly more common in Group 1 (23.5% vs. 4.9%, *p* < 0.001).

In respect to laboratory parameters, Group 1 patients demonstrated higher median serum creatinine levels (0.88 [0.36] vs. 0.79 [0.34] mg/dL, *p* = 0.049) and substantially elevated hs-cTnI levels on admission (79.3 [385.3] vs. 5.6 [8.7] pg/mL, *p* < 0.001).

The distribution of final diagnoses differed significantly between groups. In Group 1, all patients were classified within the ACS spectrum, including 31 cases of NSTEMI (91%) and 3 cases of unstable angina (9%). In contrast, Group 2 comprised a heterogeneous range of cardiovascular and non-cardiovascular conditions (Table 4). Notably, 12 patients (2.7%) in group 2 were diagnosed with ACS (10 with unstable angina and 2 with NSTEMI) but did not demonstrate significant coronary artery stenosis on subsequent coronary angiography.

Among the 46 patients assigned to Group 1, all underwent coronary angiography. Coronary angiographic findings for this group are summarized in Table 5. The extent of coronary artery disease was balanced, with single-vessel involvement identified in 16 patients (47.1%) and multivessel disease in 18 patients (52.9%). SCS predominantly involved the left anterior descending artery (LAD), identified in 32 patients (94.1%). The left circumflex artery (LCx) and right coronary artery (RCA) were affected in 16 (47.1%) and 14 (41.2%) patients, respectively. Left main artery (LMA) involvement was infrequent, present in 3 patients (8.8%).

### 3.2. HEART Score Analysis and Predictive Value for ACS with SCS

Analysis of the HEART score distribution revealed significantly higher values in Group 1 compared with Group 2 (median 8.0 [IQR 1.0] vs. 3.0 [IQR 3.0] points, *p* < 0.001). The majority of patients with ACS and SCS were classified as high risk (82.4%), whereas those in Group 2 predominantly fell within the low- and intermediate-risk categories (94.6%). Across the individual score components, Group 1 was characterized by a predominance of highly suspicious clinical history, pathological ECG changes, older age, multiple cardiovascular risk factors, and markedly elevated troponin values, all with statistically significant differences relative to Group 2 (Table 6).

**Figure 2:**
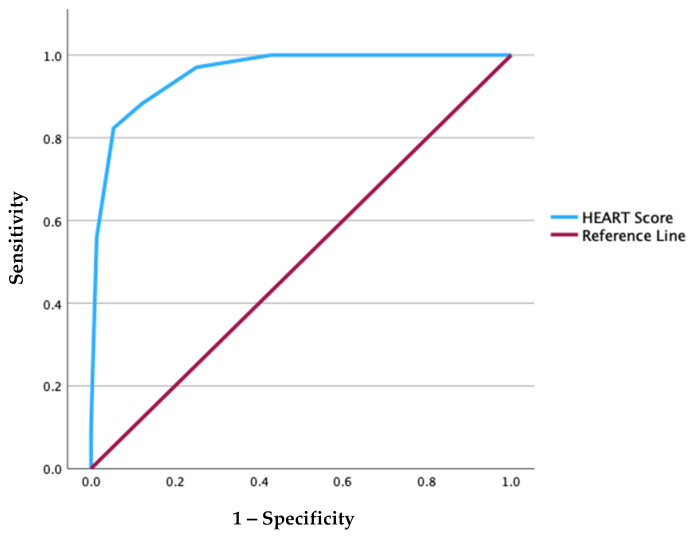
Receiver operating characteristic curve of the HEART score for acute coronary syndrome with significant coronary stenosis in the overall study population.

The discriminative performance of the HEART score for predicting ACS with SCS was evaluated using receiver operating characteristic (ROC) analysis (Figure 2). The area under the curve (AUC) was 0.956 (95% confidence interval [CI], 0.929–0.983; *p* < 0.01). A HEART score of six or higher corresponded to a sensitivity of 88.2% (95% CI: 73.4–95.3) and a specificity of 87.9% (95% CI: 84.7–90.7) for ACS with SCS detection, corresponding to the highest Youden’s index (0.761).

**Figure 3:**
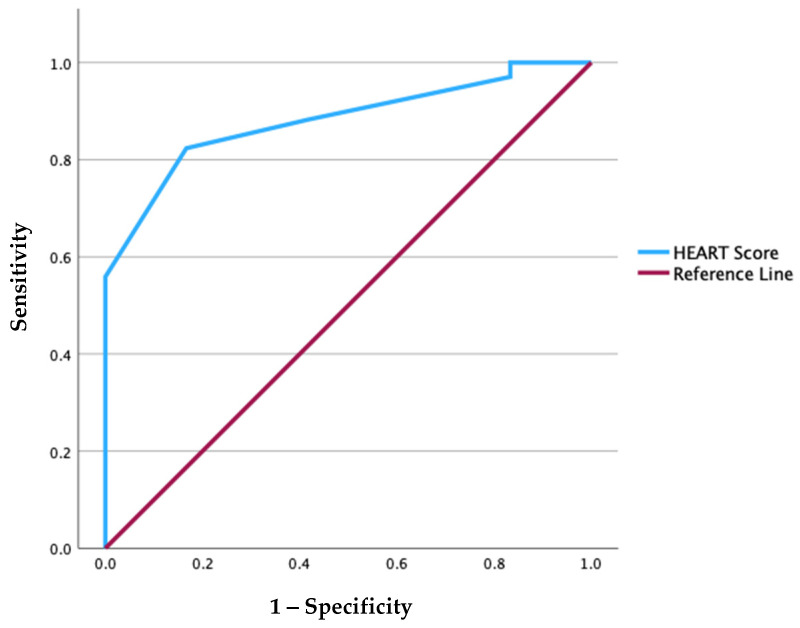
Receiver operating characteristic curve of the HEART score for significant coronary stenosis in the subgroup of patients with confirmed acute coronary syndrome.

To further evaluate the performance of the HEART score, a sensitivity analysis was performed in the subgroup of 46 patients with confirmed ACS who underwent invasive coronary angiography. ROC analysis yielded an AUC of 0.881 (95% CI: 0.784–0.978; *p* < 0.001) for the prediction of angiographically confirmed SCS (Figure 3). A HEART score of seven or higher corresponded to the highest Youden’s index (0.657), with a sensitivity of 82.0% (95% CI: 65.5–93.2%) and a specificity of 83.0% (95% CI: 51.6–97.9%).

### 3.3. Post-Discharge Outcomes and MACE Analysis

Post-discharge clinical outcomes were assessed at 6 and 12 months following the index emergency department presentation. MACE was defined as a composite outcome including acute myocardial infarction, percutaneous coronary intervention, coronary artery bypass grafting, cardiovascular mortality, and all-cause mortality. The results are summarized in Table 7.

At the 6-month follow-up, MACE occurred in 21 patients (4.4% of the total cohort), with a significantly higher incidence in patients with a HEART score ≥ 6 compared with those with a HEART score < 6 (10.7% vs. 3.0%, *p* = 0.002). At 12 months, a total of 38 patients (7.9%) experienced MACE, again with a significantly higher event rate in the HEART score ≥ 6 group (23.8% vs. 4.5%, *p* < 0.001).

## 4. Discussion

This retrospective single-center study of patients presenting to the ED with acute chest pain without ST-segment elevation demonstrates strong discriminative performance of the HEART score in predicting ACS associated with SCS. Notably, 82.4% of patients with ACS and SCS were classified as high-risk, whereas 94.6% of all other patients were classified as low- or intermediate-risk groups. Overall, these findings suggest that the HEART score may extend beyond its established prognostic role and may also reflect the presence of anatomically significant CAD.

Our findings are consistent with prior evidence evaluating the association between the HEART score and both ACS and SCS, although important differences in study design, clinical setting, and patient selection should be considered when interpreting the results. Han et al. [17], in a retrospective study of patients initially presenting to the ED and subsequently admitted to the cardiology department, evaluated a more selected inpatient population, with a high ACS prevalence (54.1%) and 30.4% of patients classified as high risk (HEART score 7–10). Similarly, Visser et al. [15], in a prospective cohort of ED patients with chest pain, included an unselected population and reported an intermediate ACS prevalence (29%), reflecting broader inclusion criteria. Arslan et al. [16], using coronary computed tomography angiography (CCTA) in patients with suspected ACS, also investigated a pre-selected population referred for imaging, with a higher likelihood of obstructive coronary disease. In this cohort, the prevalence of ACS was 12.4%, while 25.3% of patients showed >50% stenosis on CCTA. In contrast, our study reflects an unselected ED population at the initial point of care, with a substantially lower prevalence of ACS (9.6%) and a smaller proportion of high-risk patients (10.8%).

Despite differences in population characteristics, disease prevalence, and outcome definitions, the present study shows high discriminative performance of the HEART score for ACS with SCS (AUC 0.956). Han et al. [17] reported moderate accuracy for ACS (AUC 0.706) and SCS (AUC 0.737), assessed as separate endpoints. Visser et al. [15] reported an AUC of 0.810 for ACS prediction, while Arslan et al. [16] demonstrated an increasing prevalence of obstructive coronary disease on CCTA across HEART score categories. Importantly, prior studies evaluated clinical and anatomical outcomes as independent endpoints, whereas the present study used an endpoint combining ACS with angiographically confirmed SCS. This difference in endpoint definition likely contributes to the higher discriminative performance observed in the present analysis and limits direct comparability across studies.

To further evaluate the discriminatory performance of the HEART score among patients with confirmed ACS, a sensitivity analysis restricted to patients who underwent invasive coronary angiography was performed. In this subgroup, the HEART score remained a good discriminator of angiographically confirmed SCS (AUC 0.881). Although this value was lower than that observed in the overall study population, it remained higher than those reported in previous studies evaluating SCS as an isolated anatomical endpoint. These findings suggest that the discriminatory performance observed in the primary analysis reflects the intended use of the HEART score as an early triage tool in an unselected emergency department population, while the sensitivity analysis provides complementary evidence of its ability to discriminate between SCS among patients with confirmed ACS.

Several factors may explain the discriminative performance observed in our cohort. First, the outcome definition was based on ACS with SCS confirmed by invasive coronary angiography in all cases undergoing anatomical assessment. In contrast, prior studies used more heterogeneous definitions of CAD and did not consistently rely on systematic anatomical assessment across all patients, which may influence the strength of the association between the HEART score and true obstructive disease. Second, the systematic use of high-sensitivity troponin assays may have improved risk stratification within the HEART score, particularly in intermediate-risk patients, a subgroup known to be clinically heterogeneous and associated with lower discriminatory performance in previous studies, where troponin assessment strategies were more variable.

However, despite its high overall performance, the HEART score demonstrated limitations in intermediate-risk patients, consistent with prior literature. In the present cohort, 6 patients with ACS and SCS (3.5% of the intermediate-risk group) were classified as intermediate-risk (HEART score 4–6), while no cases of ACS and SCS occurred in the low-risk category, demonstrating a clear gradient of risk stratification but persistent diagnostic uncertainty within this subgroup. This subgroup therefore remains clinically challenging, as also reported by Visser et al. [15], where intermediate scores were associated with substantial overlap between patients with and without clinically significant disease, with ACS present in 27% of intermediate-risk patients. In comparison, Visser et al. [15] evaluated ACS as the study endpoint, whereas the present analysis focused on ACS with angiographically confirmed SCS. These differences in outcome definition should be considered when interpreting and comparing intermediate-risk performance across studies.

In contrast to the findings of Han et al. [17], where individual HEART score components such as clinical history and cardiovascular risk factors did not differ significantly between groups defined according to ACS status or the presence of SCS, all elements of the score in the present study demonstrated statistically significant differences between patients with and without ACS with SCS. This consistent pattern supports the robustness of the HEART score in relation to the defined endpoint and suggests a strong alignment between its individual components and the presence of clinically and anatomically significant disease.

Post-discharge outcomes were additionally evaluated as an exploratory secondary analysis at 6 and 12 months to provide complementary insight into the potential longer-term prognostic implications of the HEART score beyond its role in identifying ACS with SCS. While the HEART score has been extensively validated for short-term prediction of MACE within 30 days to 6 weeks [11], evidence regarding longer-term outcomes remains limited. In the present study, higher 6- and 12-month MACE rates were observed in patients with HEART scores ≥ 6, although these findings should be interpreted with caution given the small sample size and low number of events.

Taken together, these findings reinforce the potential role of the HEART score as a pragmatic tool for early risk stratification in patients presenting to the ED with acute chest pain. Current clinical guidelines recommend structured risk stratification using established tools such as the GRACE and TIMI risk scores, which are primarily designed to predict prognosis and guide management. These scores integrate clinical, electrocardiographic, and laboratory variables and are particularly useful following the initial diagnostic assessment. In contrast, the HEART score was developed as a rapid bedside tool for early risk stratification in unselected patients presenting with chest pain in the ED. In this context, the findings of the present study suggest that, beyond its established role in predicting short-term adverse events, the HEART score may also provide incremental value by identifying patients with ACS associated with SCS at an earlier stage of evaluation. This potential to bridge initial clinical assessment with underlying coronary anatomy may complement guideline-recommended strategies and support more timely identification of patients who may benefit from invasive evaluation.

From a practical perspective, these findings are particularly relevant in unselected ED populations, where ACS prevalence is relatively low and efficient risk stratification is essential to optimize resource utilization. The HEART score may therefore support more targeted use of coronary angiography while avoiding unnecessary invasive procedures in low-risk patients.

Future research should focus on validating these findings in larger, prospective, and multicenter cohorts to confirm reproducibility and generalizability. Further investigation should also explore the integration of the HEART score with contemporary diagnostic strategies, including serial high-sensitivity troponin algorithms and advanced imaging modalities such as CCTA. In addition, given the persistent diagnostic uncertainty observed in intermediate-risk patients, future work should aim to refine risk stratification in this subgroup through additional biomarkers or combined clinical-imaging approaches.

### Limitations

Our study has several limitations that should be acknowledged. First, the retrospective, single-center design may introduce inherent selection and information biases and limit the generalizability of the findings to other clinical settings and healthcare systems. Second, the limited number of patients with ACS and SCS may affect the precision of performance estimates and the stability of sensitivity and specificity calculations, despite the strong overall discriminative ability observed. Third, the retrospective calculation of the HEART score may have introduced measurement bias, particularly for subjective components such as clinical history and ECG interpretation. Fourth, this study assessed a combined endpoint of ACS with angiographically confirmed SCS, whereas previous studies have primarily evaluated ACS and SCS as separate outcomes, which may limit direct comparability of performance estimates. Finally, coronary angiography was performed according to clinical indication and not systematically in all patients; however, as the primary endpoint required ACS in combination with angiographically confirmed SCS, the absence of coronary angiography in patients without ACS would not have altered endpoint classification and is unlikely to have materially influenced the results. To further address this methodological aspect, a sensitivity analysis restricted to patients with confirmed ACS who underwent invasive coronary angiography was additionally performed.

## 5. Conclusions

Overall, our study demonstrates that the HEART score shows high discriminative performance for identifying patients with ACS associated with SCS in an unselected ED population, supporting its potential role in early risk stratification and in guiding clinical decision-making.

This article integrates data from two previously presented abstracts: a preliminary analysis based on a smaller cohort presented at the Portuguese Congress of Cardiology (19–21 April 2024) [18], and an expanded analysis including a larger cohort and comparison of multiple risk scores for predicting ACS with SCS presented at ESC Congress 2025, Madrid (29 August–1 September 2025) [19].

## Figures and Tables

**Table 1:** Components of the HEART score for patients with chest pain in the ED [7,8,9].

Element	Points
**Medical history ^1^**	
Highly suspicious	2
Moderately suspicious	1
Slightly suspicious	0
**ECG**	
Significant ST-depression (≥1 mm)	2
Nonspecific repolarization disturbance	1
Normal	0
**Age (years)**	
≥65	2
45–65	1
≤45	0
**Cardiovascular risk factors**	
≥3 risk factors or history of atherosclerotic disease	2
1 or 2 risk factors	1
No risk factors known	0
**Troponin**	
≥3× normal limit	2
1–3× normal limit	1
≤Normal limit	0

Scoring: 0 to 3 points = low risk; 4 to 6 points = intermediate risk; 7 to 10 points = high risk (50.1% risk). ECG—electrocardiogram; HEART—history, ECG, age, risk factors, and troponin. ^1^ Medical history is calculated based on chest pain characteristics, including high-risk features (middle/left-sided pain, diaphoresis, radiation, exertional onset, or relief with nitrates) and low-risk features (well-localized, sharp, non-exertional pain, no diaphoresis). Scores are assigned as 2 (predominantly high-risk features), 1 (mixed features), and 0 (predominantly low-risk features).

**Table 2:** Patient baseline characteristics.

	Total (*n* = 480)	Group 1 (*n* = 34)	Group 2 (*n* = 446)	*p*-Value
Male gender (*n*, %)	241 (50.2)	22 (64.7)	219 (49.1)	0.079
Age, in years (median, IQR)	59 (27.0)	66.5 (14.0)	57.5 (27.0)	**0.004**
Type 2 diabetes mellitus (*n*, %)	85 (17.7)	11 (32.4)	74 (16.6)	**0.020**
Hypertension (*n*, %)	235 (49.0)	24 (70.6)	211 (47.3)	**0.009**
Dyslipidemia (*n*, %)	203 (42.4)	25 (73.5)	178 (40.0)	**<0.001**
History of smoking (*n*, %)	40 (44.4)	8 (57.1)	32 (42.1)	0.298
Prior MI (*n*, %)	59 (12.3)	17 (50.0)	42 (9.4)	**<0.001**
Family history of CVD (*n*, %)	10 (2.1)	1 (2.9)	9 (2.3)	0.519
Atrial fibrillation (*n*, %)	54 (11.3)	3 (8.8)	51 (11.4)	1.000
HF history (*n*, %)	69 (14.4)	9 (26.5)	60 (13.5)	**0.037**
Valvular heart disease (*n*, %)	26 (5.4)	3 (8.8)	23 (5.2)	0.417
Prior ischemic stroke/TIA (*n*, %)	24 (5.0)	3 (8.8)	21 (4.7)	0.237
Peripheral arterial disease (*n*, %)	17 (3.5)	2 (5.9)	15 (3.4)	0.342
Chronic kidney disease (*n*, %)	36 (7.5)	5 (14.7)	31 (7.0)	0.164
Pulmonary disease (*n*, %)	44 (9.2)	9 (26.5)	35 (7.8)	**0.002**
Depression/anxiety (*n*, %)	154 (32.1)	7 (20.6)	147 (33.0)	0.136

CAD—coronary artery disease; CVD—cardiovascular disease; HF—heart failure; IQR—interquartile range; MI—myocardial infarction; TIA—transient ischemic attack. Statistically significant differences (*p* < 0.05) are presented in bold.

**Table 3:** Clinical and laboratory parameters.

	Total (*n* = 480)	Group 1 (*n* = 34)	Group 2 (*n* = 446)	*p*-Value
CP characteristics				
Sternal pain (*n*, %)	221 (46.0)	11 (32.4)	210 (47.1)	0.097
Epigastric pain (*n*, %)	12 (2.5)	0 (0.0)	12 (2.7)	1.000
Precordial pain (*n*, %)	243 (50.6)	23 (67.6)	220 (49.3)	**0.039**
Right-sided pain (*n*, %)	6 (1.3)	0 (0.0)	6 (1.3)	1.000
Pleuritic chest pain (*n*, %)	53 (11.0)	0 (0.0)	53 (11.9)	**0.023**
Palpation-induced CP (*n*, %)	62 (12.9)	0 (0.0)	62 (13.9)	**0.014**
Irradiation to arm/shoulder (*n*, %)	202 (42.1)	25 (73.5)	177 (39.7)	**<0.001**
Onset-to-ED time, in hours (median, IQR)	4.0 (10.0)	3.0 (6.6)	4.0 (10.0)	0.369
Accompanying symptoms				
Nausea (*n*, %)	84 (17.5)	4 (11.8)	80 (17.9)	0.361
Syncope (*n*, %)	18 (3.8)	1 (2.9)	17 (3.8)	0.797
Dyspnea (*n*, %)	47 (9.8)	2 (5.9)	45 (10.1)	0.426
Diaphoresis (*n*, %)	30 (6.3)	8 (23.5)	22 (4.9)	**<0.001**
Laboratory parameters				
Hemoglobin, g/dL (median, IQR)	14.2 (2.2)	14.4 (1.9)	14.2 (2.2)	0.679
Creatinine, mg/dL (median, IQR)	0.79 (0.34)	0.88 (0.36)	0.79 (0.34)	**0.049**
hs-cTnI, pg/mL (median, IQR)	6.1 (11.8)	79.3 (385.3)	5.6 (8.7)	**<0.001**

CP—chest pain; ED—emergency department; hs-cTnI—high-sensitivity cardiac troponin I. Statistically significant differences (*p* < 0.05) are presented in bold.

**Table 4:** Distribution of final clinical diagnoses in Group 2.

Final Clinical Diagnoses	*n* (%)
Cardiovascular disorders	114 (25.6)
Decompensated HF	29 (6.5)
Supraventricular tachycardia	7 (1.6)
Hypertensive urgency/emergency	28 (6.3)
Bradyarrhythmia	2 (0.4)
Atrial fibrillation	19 (4.3)
Pericarditis	14 (3.1)
NSTEMI	2 (0.4)
Unstable angina	10 (2.2)
Acute pulmonary embolism	3 (0.7)
No definitive diagnosis	216 (48.4)
Pulmonary disorders	19 (4.3)
Gastrointestinal disorders	26 (5.8)
Musculoskeletal disorders	40 (9.0)
Psychiatric/psychogenic disorders	31 (7.0)
Total	446 (100)

HF—heart failure; NSTEMI—non-ST-segment elevation myocardial infarction.

**Table 5:** Coronary angiography characteristics in Group 1.

	*n* (%)
CAD extent	
Single-vessel disease	16 (47.1)
Multivessel disease	18 (52.9)
SCS distribution	
LMA	3 (8.8)
LAD	32 (94.1)
LCx	16 (47.1)
RCA	14 (41.2)

CAD—coronary artery disease; LAD—left anterior descending artery; LCx—left circumflex artery; LMA—left main artery; RCA—right coronary artery.

**Table 6:** Comparison of HEART score components and total score between study groups and in the overall population.

	Total (*n* = 480)	Group 1 (*n* = 34)	Group 2 (*n* = 446)	*p*-Value
**HEART score variables**				
**Medical history**				<0.001
Highly suspicious	74 (15.4)	24 (70.6)	50 (11.2)	
Moderately suspicious	221 (46.0)	8 (23.5)	213 (47.8)	
**Slightly** suspicious	185 (38.5)	2 (5.9)	183 (41.0)	
**ECG**				<0.001
Significant ST-depression (≥1 mm)	12 (2.5)	6 (17.6)	6 (1.3)	
Nonspecific repolarization disturbance	85 (17.7)	16 (47.1)	69 (15.5)	
Normal	383 (79.8)	12 (35.3)	371 (83.2)	
**Age (years)**				0.002
≥65	184 (38.3)	20 (58.8)	164 (36.8)	
45–65	187 (39.0)	13 (38.2)	174 (39.0)	
≤45	109 (22.7)	1 (2.9)	108 (24.2)	
**Cardiovascular risk factors**				<0.001
≥3 risk factors or history of atherosclerotic disease	130 (27.1)	23 (67.7)	107 (24.0)	
1 or 2 risk factors	199 (41.5)	10 (29.4)	189 (42.4)	
No risk factors known	151 (31.5)	1 (2.9)	150 (33.6)	
**Troponin**				<0.001
<LOD	74 (1.5)	30 (88.2)	44 (9.9)	
LOD to 99th percentile URL	46 (9.6)	2 (5.9)	44 (9.9)	
>99th percentile URL	360 (75.0)	2 (5.9)	358 (80.3)	
**Total scoring—median (IQR)**	3.0 (3.0)	8.0 (1.0)	3.0 (3.0)	<0.001
Low risk, score (0–3)—***n*** (%)	255 (53.1)	0 (0)	255 (57.2)	0.020
Intermediate risk, score (4–6)—***n*** (%)	173 (36.0)	6 (17.6)	167 (37.4)	<0.001
High risk, score (7–10)—***n*** (%)	52 (10.8)	28 (82.4)	24 (5.4)	<0.001

LOD—limit of detection; URL—upper reference limit.

**Table 7:** Comparison of 6- and 12-month MACEs according to HEART score category.

	Total (*n* = 480)	HEART Score ≥ 6 (*n* = 84)	HEART Score < 6 (*n* = 396)	*p*-Value
6-month MACE * (*n*, %)	21 (4.4)	9 (10.7)	12 (3.0)	0.002
12-month MACE * (*n*, %)	38 (7.9)	20 (23.8)	18 (4.5)	<0.001

MACE—major adverse cardiovascular events. * No missing follow-up data at 6 or 12 months.

## Data Availability

The original contributions presented in this study are included in the article. Further inquiries can be directed to the corresponding author.

## Abbreviations

The following abbreviations are used in this manuscript:

ACS	Acute coronary syndrome
AUC	Area under the curve
CAD	Coronary artery disease
CCTA	Coronary computed tomography angiography
CI	Confidence interval
CP	Chest pain
CVD	Cardiovascular disease
ECG	Electrocardiogram
ED	Emergency department
HEART	History, ECG, age, risk factors, and troponin
HF	Heart failure
hs-cTnI	High-sensitivity cardiac troponin I
IQR	Interquartile range
LAD	Left anterior descending artery
LCx	Left circumflex artery
LMA	Left main artery
LOD	Limit of detection
MACE	Major adverse cardiac events
MI	Myocardial infarction
NSTEMI	Non-ST-segment elevation myocardial infarction
RCA	Right coronary artery
ROC	Receiver operating characteristic
SCS	Significant coronary artery stenosis
STEMI	ST-segment elevation myocardial infarction
TIA	Transient ischemic attack
URL	Upper reference limit
